# Cluster analysis to identify the profiles of individuals with compromised bone health versus unfortunate wrist fractures within the Canadian Longitudinal Study of Aging (CLSA) database

**DOI:** 10.1007/s11657-023-01350-7

**Published:** 2023-12-01

**Authors:** Joshua I. Vincent, Joy C. MacDermid, Carol W. Bassim, Pasqualina Santaguida

**Affiliations:** 1 School of Physical Therapy, University of Western Ontario, 1151 Richmond St, London, ON N6A 3K7 Canada; 2 Roth│McFarlane Hand and Upper Limb Centre, St. Joseph’s Hospital, 268 Grosvenor St, London, ON N6A 4V2 Canada; 3 School of Rehabilitation Science, McMaster University, 1280 Main St W, Hamilton, ON L8S 4L8 Canada; 4 Department of Research Methods, Evidence & Impact, McMaster University, 1280 Main St W, Hamilton, ON L8S 4L8 Canada; 5 Dept. of Medicine, McMaster University, 1280 Main St W, Hamilton, ON L8S 4L8 Canada

**Keywords:** Aging, DRF, CLSA, Cluster analysis, Bone health profiles

## Abstract

**Summary:**

We used cluster analysis to determine the profiles of individuals who sustained wrist fractures. We found two groups: (1) young and active and (2) older and less active. This information may be used to identify individuals who require further bone health interventions to optimize healthy aging.

**Introduction:**

Distal radial fractures (DRF) are the most common of all fractures, with 6% of males and 33% of females having one at some point in their lifetime. We hypothesize that DRF consists of two subpopulations: one with compromised bone health that is early in the osteoporosis (OP) trajectory and another which are active and healthy and suffer a misfortune fracture due to their high activity levels or risk-taking behaviors. The latter is likely to recover with a minimal disability, while the former may signal a negative health trajectory of disability and early mortality.

**Objective:**

To determine the profiles of individuals who sustained wrist fractures using cluster analysis within the Comprehensive Cohort of the Canadian Longitudinal Study on Aging (CLSA) database considering factors that reflect bone health and activity levels.

**Methods:**

We included all the individuals who had a wrist fracture within the CLSA comprehensive cohort of the database (*n* = 968). The baseline data was used for this analysis. A 2-step cluster analysis was used to identify profiles that were both statistically and clinically meaningful. Variables that were used in the cluster analysis include demographic variables, physical activity status indicators, general health indicators, mobility indicators, bone health indicators, comorbid conditions, and lifestyle factors.

**Results:**

We were able to identify two distinct profiles that were statistically and clinically meaningful confirming our hypothesis. One cluster included a predominantly younger cohort, who are physically active, with less comorbid conditions, better bone health, and better general health, while the opposite was true of the first cohort.

**Conclusion:**

We were able to identify two clusters—a healthy profile and a bone health compromised profile. This information may be used to identify the subgroup of people who should be targeted in the future for more intensive preventive health services to optimize healthy aging.

## Introduction

Distal radius fractures (DRF) are the most common of all fractures [1–4], with 6% of males and 33% of females [5, 6] sustaining one within their lifetime. About 40% of osteoporotic fractures in women between the ages of 50 and 60 years are DRF [7]. The incidence of DRF in men is lower and spikes later in life [7]. Upper extremity fractures can occur because of compromised bone health, or in healthy people due to misfortune. DRF is generally classified as fragility fractures when they occur due to low trauma such as a fall from a standing height. However, this is a gross generalization that does not precisely differentiate those with compromised bone health from those who have suffered misfortune.

Since DRF occurs early in the potential trajectory of osteoporosis (OP), clinical information such as bone mineral density (BMD) testing is rarely available to assist with early decision-making. Furthermore, a meta-analysis of 90,000 person-years and over 2000 fractures indicated that there is a wide overlap in the BMD of patients who develop a fracture and those who do not [8]. One Canadian population-based study of over 260,000 1st incident upper extremity fractures identified that DRF was the most frequent fracture and occurs most commonly in women in the 50–65-year-old age group. Another Canadian population-based study identified the 10-year probability of a secondary fracture after an initial DRF (14%) is elevated compared to the 10% population risk but was a lower risk indicator than the 24% rate that occurred for proximal humerus fractures (PHF) [9]. This suggests that PHF is a more definite fragility fracture than DRF. Current systems for establishing future fracture risk provide substantially different risk estimates, especially for people under 65 years of age [9]. However, what is clear across studies is that BMD alone is insufficient to characterize bone health or the risks of fracture.

Upper extremity fragility fractures occur sufficiently in advance of more osteoporotic fractures affecting the spine and larger bones, such that intensive early interventions to improve bone health along with proper nutrition and exercise are likely to have a positive impact on future bone health. Furthermore, early risk identification is a focus in current osteoporosis (OP) clinical practice guidelines [10]. Targeting secondary prevention to those at risk may prevent the severe disability and early mortality [11] that results from osteoporotic spinal and hip fractures [10]. We hypothesize that DRF consists of two subpopulations: one with compromised bone health that is early in an OP trajectory and another which consists of individuals who are active and healthy but suffer a misfortune fracture due to their lifestyle behaviors. The latter is likely to recover with minimal disability [12, 13], while the former may signal a negative health trajectory of disability and early mortality [14]. Some epidemiological data support our hypothesis of different profiles with different fracture populations since DRF occurs in more active people and is more related to weather and activity [15].

In Canada, OP costs about $2 billion/year, and those costs double if the proportion of those placed in long-term care as a result of OP is taken into consideration [16]. Previous research suggests that in older adults who sustain a fragility fracture, future healthcare costs increase exponentially post-fracture [16, 17, 17, 18]. The majority of the costs of managing OP relate to managing the incident costs of the fractures, suggesting that secondary fracture prevention is critical [16, 19]. However, given the high incidence of DRF and moderate risks, broad-scale prevention strategies that are not appropriately targeted will not be cost-effective or feasible. In addition, practice pattern analysis indicates that therapists [20] and orthopedic surgeons [21] rarely initiate early conservative bone health interventions following a DRF. Clinical indicators that could be used to appropriately target this for early intervention to a smaller subset of patients might be cost-effective and feasible for implementation.

The purpose of this study is to determine the profiles of individuals with compromised bone health and unfortunate wrist fractures using cluster analysis within the Canadian Longitudinal Study on Aging (CLSA) database.

## Methods

### *Participants*

Both men and women (*N* = 968) aged 45 and older who have had a wrist fracture and enrolled in the Comprehensive Cohort of the Canadian Longitudinal Study of Aging (CLSA) (https://www.clsa-elcv.ca/) were included in the study. The CLSA is a large long-term (20 years) national longitudinal study conducted to identify ways to help people live longer and live well and to better understand healthy aging [22, 23]. CLSA is a strategic initiative of the Canadian Institutes of Health Research (CIHR). The CLSA comprises 51,338 participants who were aged between 45 and 85 years when recruited (2010 and 2015). They will be followed up until 2033 or death whichever is earlier. Out of the 51,338 participants, 31,097 participants (comprehensive cohort) underwent in-home interviews and in-person comprehensive assessments at the data collection centers. More in-depth information can be found on the CLSA website (https://www.clsa-elcv.ca/). The baseline data from the Comprehensive Cohort was used in this study. Demographic characteristics of the wrist fracture cohort are included in Table 1.

**Table 1:** Participant characteristics

Characteristics		*n* (%)
Age group	45–54	102 (10.5)
	55–64	283 (29.2)
	65–74	294 (30.4)
	75 +	289 (29)
Sex	Female	682 (70.5)
	Male	286 (29.5)
T score neck of hip DXA	Osteoporosis	135 (13.9)
	Osteopenia	276 (28.5)
	Normal	504 (52.1)
T score WB DXA	Osteoporosis	135 (13.9)
	Osteopenia	276 (28.5)
	Normal	460 (47.5)
BMI category	Obese	275 (28.4)
	Overweight	373 (38.5)
	Underweight	14 (1.4)
	Normal	296 (30.6)
Timed Up and Go	Other	692 (71.5)
	Lowest 20%	258 (26.7)
Balance	Other	642 (66.3)
	Lowest 20%	250 (25.8)
Osteoporosis	Yes	276 (28.5)
	No	679 (70.1)
Heart diseases	Yes	135 (13.9)
	No	830 (85.7)
General health	Fair/poor	102 (10.5)
	Other	863 (89.2)
General mental health	Fair/poor	66 (6.8)
	Other	900 (93.0)
Visual self-rated	Fair/poor	94 (9.7)
	Other	873 (90.2)
	No	950 (95.1)
Osteoarthritis (of 4 diagnoses)	None	505 (52.2)
	1	269 (27.8)
	> 2	151 (15.6)
Rheumatoid arthritis	Yes	46 (4.8)
	No	908 (93.8)
Smoking	Current	73 (7.5)
	No	458 (47.3)
	Not current	437 (45.1)
Light sports	Often	57 (5.9)
	< Often	868 (89.7)
Moderate sports	Often	21 (2.2)
	< Often	904 (93.4)
Strenuous sports	Often	69 (7.1)
	< Often	856 (88.4)
Exercise	Often	84 (8.7)
	< Often	840 (86.8)
Walking	Often	475 (49.1)
	< Often	449 (46.4)

### Variables used in this study

We view health as an interaction between body structure, impairments, activity, and participation; modified by personal and environmental factors;^22^ consistent with how health and functioning are defined by the World Health Organization (WHO) International Classification of Functioning (ICF) [24]. The following variables were used in the cluster analysis:

#### Demographic variables:

age, gender, and age group [25]

#### Biological variables:

body mass index, total body fat percentage, DEXA bone scan bone mineral density (BMD) hip, and average grip strength

#### Physical activity and mobility indicators

average balance, Timed Up and Go test, and following questions from the Physical Activities (PA2) [26]. Questionnaire was used to indicate physical activity levels:

- Light sports, or recreation activities in the past 7 days (in days)
- Moderate sports, or recreation activities in the past 7 days (in days)
- Strenuous sports, or recreation activities in the past 7 days (in days)
- Strength and endurance exercise in the past 7 days (in days)
- Walking in the past 7 days (in days)
- Sitting activities in the past 7 days (in days)
- Past 7 days, light housework (yes/no)
- Past 7 days, heavy housework (yes/no)
- Past 7 days, caring for others (yes/no)

#### General health indicators

Number of falls in the past 12 months, self-rated general health, self-rated vision, grouped musculoskeletal (MSK) comorbid conditions, osteoporosis diagnosis, menopause [25]

#### Lifestyle factors

calcium supplement use, vitamin D supplement use, hormone replacement therapy (HRT), smoking [25].

### Statistical analysis

#### Data preparation

All analyses were completed using SPSS version 27(SPSS Inc., Chicago, IL). Missing data were handled using multiple imputations [27]. Missing data patterns were analyzed before completing imputations. In the baseline dataset, 23.81% of the cases had missing values and on the whole 2.7% of the data were missing. All variables except for one had missing values. Missing value pattern analysis indicated that there was no specific pattern.

#### Cluster analysis

We completed a 2-step cluster analysis of sex-stratified wrist fracture participants. We stratified by sex as we did not want the clustering to be driven by the underlying differences between the two sexes in many variables that were included in the model like body mass index (BMI), total body fat percentage, DEXA bone scan BMD hip, and average grip strength. Effectively, we completed two different 2-step cluster analyses for the following groups: (1) women who have sustained a wrist fracture and (2) men who have sustained a wrist fracture. We used the 2-step cluster analysis approach as it efficiently handles continuous and categorical variables [28]. The following variables were used in the cluster analysis: age, DEXA total body fat percentage, DEXA bone scan neck of femur T score, DEXA bone scan whole body T score, maximum grip strength, balance scores, and Timed Up and Go (TUG) test. The statistical analysis runs a pre-clustering in the first step to identify groupings and in the second step, it runs a hierarchical clustering method that automatically selects the number of clusters. For the second step, the program utilizes the Bayesian information criterion (BIC) to select the “best” cluster solution. A smaller BIC value indicates a better-fitting model. The 2-step cluster analysis uses a distance measure that defines the distance between two clusters as the corresponding decrease in log-likelihood by combining them into one cluster [29, 30]. It can handle large datasets with ease because of the pre-clustering completed in the first step.

## Results

### Sample characteristics

Women comprised over two-thirds of the participants (70.5%). The mean age was 67.5 years SD 9.72 with a range from 45 to 84 years. About 28.5% and 13.9% of the participants were diagnosed with osteopenia and osteoporosis, respectively. Two-thirds of the participants (66.9%) were either obese or overweight. Most participants (89.2%) rated their general health as good or excellent. Forty-three percent of the participants had an osteoarthritis diagnosis. The majority of participants rated themselves as active on the physical activity self-report questions (see Table 1 for details).

### Cluster analysis

For both sexes, a two-step cluster analysis identified 2 distinct clusters with homogenous patterns of the variables that were included in the model. We named the first cluster as younger and active while the second cluster was named older and less active.

#### Wrist fracture group (female) (seeTables 2, 3, and 4)

**Table 2:** Cluster characteristics within the wrist fracture group for demographic and physiological variables stratified by gender

Characteristic	Female		Male	
	Cluster 1—younger/active group (*n* = 246)	Cluster 2—older/less active group (*n* = 279)	Cluster 1—younger/active group (*n* = 112)	Cluster 2—older/less active group (*n* = 129)
Age—mean (SD)	61.5 (7.79)	73.13 (7.11)	59.72 (8.76)	69.21 (8.13)
Age group—*n* (%)				
45–54 55–64 65–74 75 +	48 (19.5) 114 (46.3) 69 (28.0) 15 (6.1)	2 (.7) 35 (12.5) 95 (34.1) 147 (52.7)	34 (30.4) 49 (43.8) 21 (18.8) 8 (7.1)	6 (4.7) 34 (26.4) 52 (40.3) 37 (28.6)
Menopause—*n* (%)				
Yes No	214 (87.0) 30 (12.2)	268 (96.1) 11 (3.9)	NA	NA
HRT use—*n* (%)				
Yes No	91 (37.0) 155 (63.0)	143 (51.3) 134 (48.0)	NA	NA
DEXA femoral neck T score—mean (SD)	− 1.35 (1.06)	− 1.82 (0.84)	− 0.41 (1.14)	− 0.89 (1.01)
Hip T score classification—*n* (%)				
Osteoporosis Osteopenia Normal	27 (11.0) 133 (54.1) 82 (33.3)	59 (21.1) 166 (59.5) 49 (17.6)	1 (.9) 33 (29.5) 78 (69.6)	4 (3.1) 58 (45.0) 62 (48.1)
DEXA whole body T score—mean (SD)	− 0.97 (1.37)	− 1.62 (1.30)	0.73 (1.34)	0.25 (1.40)
Whole body T score classification—*n* (%)				
Osteoporosis Osteopenia Normal	35 (14.2) 87 (35.4) 124 (50.4)	75 (26.9) 129 (46.2) 75 (26.9)	1 (.9) 9 (8.0) 102 (91.1)	2 (1.6) 16 (12.4) 111 (86.0)
DEXA total body fat percentage—mean (SD)	38.69 (5.84)	40.71 (5.87)	25.94 (4.47)	30.82 (5.26)
BMI classification—*n* (%)				
Obese Overweight Underweight Normal	57 (23.2) 88 (35.8) 4 (1.6) 97 (39.4)	72 (25.8) 114 (40.9) 6 (2.2) 87 (31.2)	25 (22.3) 53 (47.3) 34 (30.4)	50 (38.8) 56 (43.4) 23 (17.8)

**Table 3:** Cluster characteristics within the wrist fracture group for lifestyle and general health variables stratified by gender

Characteristic	Female		Male	
	Cluster 1—younger/active group (*n* = 246)	Cluster 2—older/less active group (*n* = 279)	Cluster 1—younger/active group (*n* = 112)	Cluster 2—older/less active group (*n* = 129)
Smoking category—*n* (%)				
Yes (I currently smoke) No Former	16 (6.5) 125 (50.8) 105 (42.7)	15 (5.4) 145 (52.0) 119 (42.7)	10 (8.9) 56 (50.0) 46 (41.1)	14 (10.9) 45 (34.9) 70 (54.3)
Self-reported Gen health—*n* (%)				
Excellent Very good Good Fair Poor	56 (22.8) 116 (47.2) 61 (24.8) 12 (4.9) 1 (.4)	41 (14.7) 115 (41.2) 90 (32.3) 25 (9.0) 7 (2.5)	29 (25.9) 54 (48.2) 24 (21.4) 3 (2.7) 2 (1.8)	17 (13.2) 56 (43.4) 39 (30.2) 11 (8.5) 4 (3.1)
Self-reported vision—*n* (%)				
Excellent Very good Good Fair Poor	54 (22.0) 108 (43.9) 68 (27.6) 12 (4.9) 3 (1.2)	49 (17.6) 90 (32.3) 105 (37.6) 27 (9.7) 8 (2.9)	26 (23.2) 51 (45.5) 27 (24.1) 8 (7.1) 0 (0)	26 (20.2) 53 (41.1) 38 (29.5) 10 (7.8) 2 (1.6)
Osteoporosis—*n* (%)				
Yes No	70 (28.5) 173 (70.3)	123 (44.1) 151 (54.1)	4 (3.6) 107 (95.5)	14 (10.9) 114 (88.4)
Heart disease—*n* (%)				
Yes No	13 (5.3) 231 (93.9)	48 (17.2) 230 (82.4)	8 (7.1) 104 (92.9)	27 (20.9) 102 (79.1)
Osteoarthritis—*n* (%)				
Yes No	96 (39) 140 (56.9)	133 (47.7) 129 (46.2)	23 (20.6) 88 (78.6)	49 (38) 77 (59.7)
Rheumatoid arthritis—*n* (%)				
Yes No	13 (5.3) 230 (93.5)	13 (4.7) 260 (93.2)	1 (0.9) 111 (99.1)	3 (2.3) 123 (95.3)
Multivitamin use—*n* (%)				
Yes No	80 (32.5) 159 (64.6)	103 (36.9) 163 (58.4)	17 (15.2) 91 (81.3)	38 (29.5) 83 (64.3)
Vitamin D use—*n* (%)				
Yes No	162 (65.9) 77 (31.3)	199 (71.3) 67 (24.0)	45 (40.2) 63 (56.3)	59 (45.7) 64 (49.6)
Calcium use—*n* (%)				
Yes No	124 (50.4) 115 (46.7)	143 (51.3) 123 (44.1)	35 (31.3) 74 (66.1)	30 (23.3) 92 (71.3)

**Table 4:** Cluster characteristics within the wrist fracture group for physical performance measures and physical activity variables derived from PASE stratified by gender

Characteristic	Female		Male	
	Cluster 1—younger/active group (*n* = 246)	Cluster 2—older/less active group (*n* = 279)	Cluster 1—younger/active group (*n* = 112)	Cluster 2—older/less active group (*n* = 129)
Maximum grip strength—mean (SD)	27.33 (4.98)	22.13 (5.11)	48.16 (7.62)	37.72 (8.05)
TUG time—mean (SD)	8.74 (1.19)	10.51 (2.06)	8.65 (1.22)	10.19 (1.79)
Chair Rise test—mean (SD)	12.94 (3.21)	14.99 (3.84)	12.13 (3.19)	14.46 (3.91)
Balance scores—mean (SD)	48.20 (17.36)	9.17 (9.52)	53.59 (13.32)	15.49 (17.51)
Strenuous sports—*n* (%)				
Never Seldom (1 to 2 days) Sometimes (3 to 4 days) Often (5 to 7 days)	140 (56.9) 41 (16.7) 36 (14.6) 22 (8.9)	203 (72.8) 29 (10.4) 20 (7.2) 14 (5.0)	58 (51.8) 19 (17.0) 22 (19.6) 10 (8.9)	87 (67.4) 13(10.1) 12 (9.3) 11 (8.5)
Moderate sports—*n* (%)				
Never Seldom (1 to 2 days) Sometimes (3 to 4 days) Often (5 to 7 days)	204 (82.9) 20 (8.1) 7 (2.8) 8 (3.3)	235 (84.2) 23 (8.2) 7 (2.5) 1 (.4)	91 (81.3) 8 (7.1) 7 (6.3) 3 (2.7)	110 (85.3) 7 (5.4) 2 (1.6) 4 (3.1)
Light sports—*n* (%)				
Never Seldom (1 to 2 days) Sometimes (3 to 4 days) Often (5 to 7 days)	174 (70.7) 31 (12.6) 13 (5.3) 21 (8.5)	214 (76.7) 29 (10.4) 10 (3.6) 13 (4.7)		103 (79.8) 12 (9.3) 4 (3.1) 4 (3.1)
Strengthening exercises—*n* (%)				
Never Seldom (1 to 2 days) Sometimes (3 to 4 days) Often (5 to 7 days)	159 (64.6) 28 (11.4) 39 (15.9) 13 (5.3)	197 (70.6) 23 (8.2) 27 (9.7) 19 (6.8)	67 (59.8) 11 (9.8) 20 (17.9) 11 (9.8)	80 (62.0) 15 (11.6) 11 (8.5) 16 (12.4)
Walking frequency—*n* (%)				
Never Seldom (1 to 2 days) Sometimes (3 to 4 days) Often (5 to 7 days)	31 (12.6) 31 (12.6) 54 (22.0) 123 (50.0)	52 (18.6) 43 (15.4) 43 (15.4) 128 (45.9)	12 (10.7) 9 (8.0) 23 (20.5) 65 (58.0)	16 (12.4) 20 (15.5) 18 (14.0) 69 (53.5)
Heavy housework—*n* (%)				
Yes No	180 (73.2) 59 (24.0)	167 (59.9) 99 (35.5)	80 (71.4) 29 (25.9)	70 (54.3) 53 (41.1)
Home repairs—*n* (%)				
Yes No	30 (12.2) 209 (85.0)	25 (9.0) 241 (86.4)	41 (36.6) 68 (60.7)	45 (34.9) 78 (60.5)
Yard work—*n* (%)				
Yes No	113 (45.9) 126 (51.2)	83 (29.7) 183 (65.6)	72 (64.3) 37 (33.0)	69 (53.5) 54 (41.9)

##### Younger and active cluster (***n*** = 246, 46.9%)

They were relatively younger with a mean age of 63.1 years when compared to the older and less active cluster (mean age of 73.13 years). The majority fell within the 45 to 64 age group (66%). Most had gone through menopause (87%). Thirty-seven percent of them were treated with hormone replacement therapy (HRT). These participants had better T scores when compared to the older and less active cluster (femoral neck—mean − 1.35; SD 1.06 and whole body—mean − 0.97; SD 1.37). The majority (59%) were either obese or overweight. They reported that they have very good or excellent general health (70%) and vision (66%). Most were either non-smokers (51%) or former smokers (43%). They reported a relatively high level of physical activity when compared to the other cluster. Almost half of them reported taking part in strenuous sports activities (40%); almost everyone walked in the last week (87%); close to three-quarters reported doing heavy housework (73%); and 46% did some yard work. This cluster faired well in the physical performance tests when compared to the other cluster. They had higher grip strength (mean 27.33 kg; SD 4.98); higher balance scores (mean 48.20 s; SD 17.36); a lower TUG time (mean 8.74 s; SD 1.19); and a lower chair rise test time (mean 12.94 s; SD 3.21).

##### Older and less active cluster (***n*** = 279, 53.1%)

This group was relatively older with a mean age of 73.13 years (SD 7.11) when compared to the younger and active cluster. The majority fell within the 65 and older age group (87%). Most had gone through menopause (96%). Fifty-one percent were treated with HRT. They had lower T scores when compared to the other cluster (femoral neck—mean − 1.82; SD 0.84 and whole body—mean − 1.62; SD 1.30) and forty-four percent were osteoporotic. More than two-thirds of the participants were either obese or overweight. More than half of them reported that they have very good or excellent general health (56%) and vision (50%). Most of them were either non-smokers (52%) or former smokers (43%). They reported relatively lower levels of physical activity when compared to the other cluster. Almost one-quarter of them reported taking part in strenuous sports activities (23%); three-quarters reported that they walked in the last week (77%); 59% did heavy housework; and 30% did some yard work. This cluster performed poorly in the physical performance tests when compared to the other cluster. They had lower grip strength (mean 23.13 kg; SD 5.11); lower balance scores (mean 9.17 s; SD 9.52); a higher TUG time (mean 10.51 s; SD 2.06); and a higher chair rise test time (mean 14.99 s; SD 3.84).

#### Wrist fracture group (male) (seeTables 2, 3, and 4)

##### Younger and more active cluster (***n*** = 112, 46.5%)

They were relatively younger with a mean age of 59.72 years (SD 8.76) when compared to the other cluster. The majority fell within the 45 to 64 age group (74%). They had better T scores when compared to the younger and active cluster (femoral neck—mean − 0.41; SD 1.14 and whole body—mean 0.73; SD 1.34). Only 4% were osteoporotic. Seventy percent of them were either obese or overweight. Most reported that they had very good or excellent general health (74%) and vision (69%). Most of them were either non-smokers (50%) or former smokers (41%). They reported a relatively high level of physical activity when compared to the other cluster. Almost half of them reported taking part in strenuous sports activities (48%); almost everyone walked in the last week (89%); close to three-quarters reported doing heavy housework (71%); and 64% did some yard work. This cluster faired well in the physical performance tests when compared to the older and less active cluster. They had higher grip strength (mean 48.16 kg; SD 7.62); higher balance scores (mean 53.59 s; SD 13.32); a lower TUG time (mean 8.65 s; SD 1.22); and a lower chair rise test time (mean 12.13 s; SD 3.19).

##### Older and less active cluster (***n*** = 129, 53.5%)

They were relatively older with a mean age of 69.21 years (SD 8.13) when compared to the other cluster. Most of them fell within the 65 and older age group (69%). They had lower T scores when compared to the younger and active cluster (femoral neck—mean − 0.89; SD 1.01 and whole body—mean 0.25; SD 1.40). Eleven percent were osteoporotic. More than three-quarters of them were either obese or overweight. More than half of them reported that they have very good or excellent general health (57%) and vision (61%). Most of them were either non-smokers (35%) or former smokers (54%). They reported relatively lower levels of physical activity when compared to the other cluster. More than a quarter of them reported taking part in strenuous sports activities (28%); 88% reported that they walked in the last week; 54% did heavy housework; and 54% did some yard work. This cluster performed poorly in the physical performance tests when compared to the other cluster. They had relatively lower grip strength (mean 37.72 kg; SD 8.05); lower balance scores (mean 15.49 s; SD 17.51); a higher TUG time (mean 10.19 s; SD 1.79); and a higher chair rise test time (mean 14.46 s; SD 3.91).

## Discussion

The results of the current study support our hypothesis that within those who sustain distal wrist fractures, there are two distinct groups of individuals. One group is characterized by older individuals with lower BMD and lower physical activity levels. This is consistent with age-related changes exacerbated by inactivity and poor nutrition [31] that may result in compromised bone health and true fragility fractures. The other cluster is characterized by younger individuals, with normal bone mineral density and higher physical activity levels; in this group, fractures are more likely attributable to misfortunate while engaged in physical activity. These separate subgroups explain why DRF is less predictive of future fractures than PHF [32] which occurs in an older more fragile population. This study suggests that DRF has subtypes of fragility fracture and misfortunate fracture. This has clinical relevance since these subgroups have different risk trajectories and may have different expectations for functional recovery.

Frailty is an important medical and public health problem [33] that can result in an increased burden on the healthcare system in terms of utilization and cost [34, 35]. Frailty comprises a combination of clinical and physiological changes in the human body as individuals grow older. In the current study, the older and less active cluster with distal wrist fracture was characterized by the presence of decreased grip strength, longer time duration to complete the chair rise test, and lower physical activity. These characteristics match the phenotype for frailty proposed by previous studies [33, 36–38]. Since the incidence of a DRF is not a stand-alone indicator of frailty, these cluster characteristics must be considered. However, a DRF should act as a positive screening test, indicating the need to assess frailty parameters and identify aspects that can be remediated.

The older and compromised bone health cluster should be the target group for interventions related to bone health, such as fall prevention strategies and exercise-based programs aimed at increased strength, balance, and endurance. A study where post-menopausal women were followed up for more than 11 years concluded that a wrist fracture is associated with increased risk of the subsequent hip (HR = 1.36, 95% CI 1.26–1.48), spine (HR = 1.48, 95% CI 1.32–1.66), upper extremity (HR = 1.88, 95% CI 1.70–2.07), and lower extremity fractures (HR = 1.36, 95% CI 1.26–1.48) [39]. If these interventions are not applied at the right time, it can be considered as a missed opportunity to contain a cascade of fractures that are non-wrist related [20]. However, previous research described below indicates that these interventions are less often initiated with wrist fractures. A retrospective nationwide cohort study in Korea concluded that wrist fracture patients were less often evaluated and treated for osteoporosis by their treating physician managing the fracture when compared to hip or spine fractures [40]. A Canadian study indicated that only 50% of those with a wrist fracture were followed up for osteoporosis evaluation and management post-fracture [41]. Patients presenting with wrist fractures with the characteristics described in this study must be screened for osteoporosis and utilize this as a window of opportunity to put in place targeted interventions to prevent further fractures and resulting frailty and disability [11].

A previous study demonstrated younger patients with DRF are at greater risk of poor outcomes in terms of pain and functional disability when malalignment of the fracture is present, compared to older patients [42]. This is presumed to be related to life demands, a concept that is supported in our cluster findings. However, studies also suggest that the younger active cohort might recover faster than the older adult group [43]. Young women with a wrist fracture are more prone to subsequent non-wrist fractures [39]. In addition, the management of wrist fractures in this group differs from the elderly frail group [44]. However, clinical practice guidelines [45, 46] recommend a bone mineral density test in the following cases: in all women over 65 and men over 70 years of age; individuals who break a bone after age 50; a woman of menopausal age with risk factors; and postmenopausal woman under age 65 with risk factors. This recommendation unfortunately does not address women less than 50 years of age who sustain a wrist fracture. This might affect the prospects of this cohort of women obtaining a BMD scan, which can potentially help prevent secondary fractures.

The younger and active group reported higher engagement in moderate to strenuous activities suggesting that to a certain extent, the fracture risk may have been related to activities, environmental hazards, and risk-taking. Secondary fracture prevention for this group might focus on fall hazard reduction and activity analysis. Since the younger more active cohort fracture profiles were less representative of fragility fractures, we hypothesize they may have occurred with high-energy trauma and may require more specialized fracture management to optimize anatomic reduction and allow individuals to return to their previously active lifestyle.

Our study showed that individuals who were in the older and less active group also had relatively lower levels of bone mineral density and higher rates of osteoporosis diagnosis. A previous study has found that a decrease of 1 SD of the femoral neck BMD was associated with a 66% higher relative risk of wrist fracture [47]. In addition, we also found that there was a greater incidence of osteoporosis and lower BMD in women than in men in both groups. These study findings are in line with previous estimates [48–51] that indicate a strong female predominance among those incurring a DRF, especially after the age of 50.

**Fig. 1:**
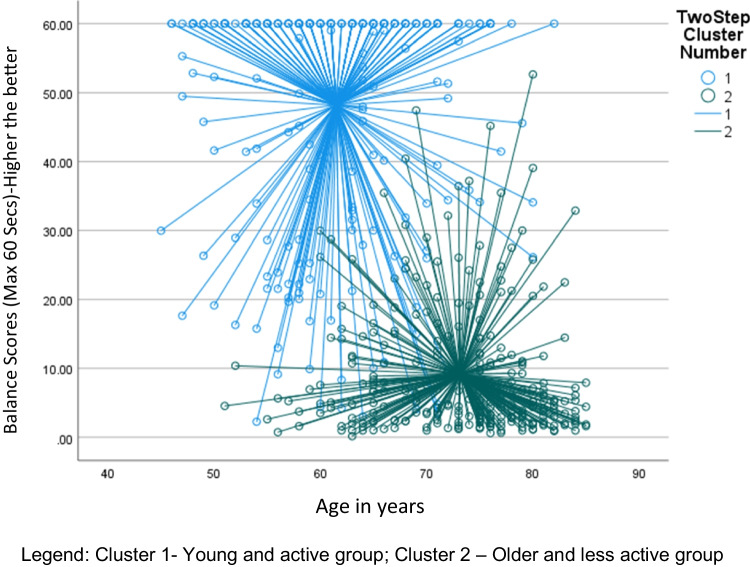
Scatter plot showing the distribution of balance scores plotted against age between the clusters for females within the wrist fracture group. Legend: cluster 1—young and active group; cluster 2—older and less active group

**Fig. 2:**
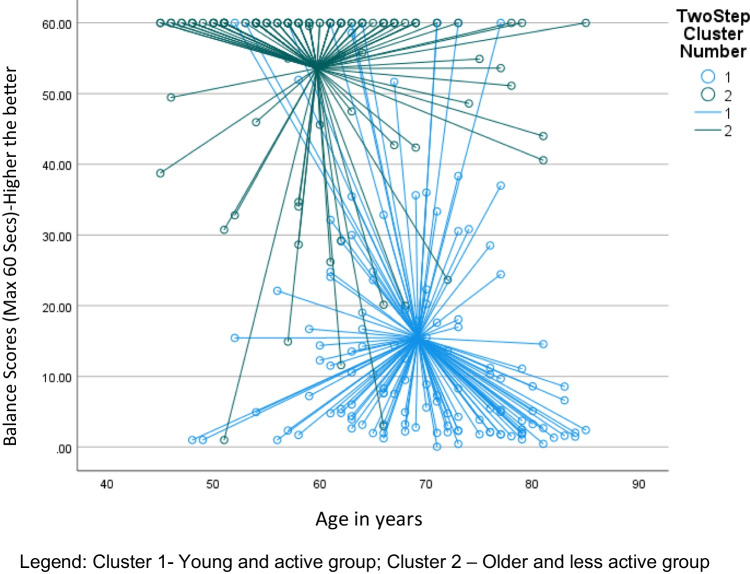
Scatter plot showing the distribution of balance scores plotted against age between the clusters for males within the wrist group. Legend: cluster 1—young and active group; cluster 2—older and less active group

The balance score was the main indicator in differentiating the clusters in the 2-step cluster analysis in the current study. This did not come as a surprise as previous studies have confirmed that balance levels decline in individuals as they age [52, 53]. A review completed to look into the changes in gait and balance associated with aging found that older adults showed an age-related decrease in balance and increased gait variability with associated risk for falls [53]. This may explain why this factor turned out to be the significant factor in differentiating the clusters. Another significant indicator was grip strength. Grip strength as an indicator of general health [54] has been previously shown that the unaffected side grip strength can predict fracture risk in the 4 years after a DRF in a prospective cohort study [55]. Another study has concluded that the grip strength of the unaffected side can act as a surrogate for general bone health, frailty, and overall muscle strength [20]. We found stark differences in both the balance scores and grip strength between the two clusters (Figs. 1 and 2). The older less active cluster demonstrated poorer balance and grip strength. This is in line with previous studies. A case–control study identified that older adults with distal radius fractures (DRF) had poorer balance [56]. In another case–control study, women with DRF had poorer dynamic balance and grip strength [57]. Studies have indicated that exercise intervention focussed on strength and balance is effective for fracture prevention in the elderly [58, 59]. However, the implementation rate of these interventions is very low [56].

The strengths of the current study include the use of data from a large population-based database that was randomly sampled. This has helped us to study the profiles of people from different geographical regions and social strata in Canada. This increases the power and generalizability of our findings. Our study also had some limitations. A limitation common to all CLSA studies is that this large population study may reflect a healthier volunteer cohort rather than the true population norms. This could be because participation in the current study would have been more difficult for those with mobility, health, or transportation issues since it required in-person testing. Another limitation is that the information about fracture is limited to self-reported, is not time-indexed, and we have limited information on the nature of the fracture or its management.

In conclusion, this study was able to identify two distinct clusters with profiles of people who sustain a wrist fracture—an older less active cluster and a younger active group. Screening procedures and secondary prevention interventions should be tailored to these different risk profiles with the type and weighting of hazard awareness, balance training, and strength training.

## Data Availability

Data are available from the Canadian Longitudinal Study on Aging (www.clsa-elcv.ca) for researchers who meet the criteria for access to de-identified CLSA data.

## Declarations

### Disclaimer

The opinions expressed in this manuscript are the author’s own and do not reflect the views of the Canadian Longitudinal Study on Aging.
